# Dulaglutide exerts beneficial anti atherosclerotic effects in ApoE knockout mice with diabetes: the earlier, the better

**DOI:** 10.1038/s41598-020-80894-x

**Published:** 2021-01-14

**Authors:** Junpei Sanada, Atsushi Obata, Yoshiyuki Obata, Yoshiro Fushimi, Masashi Shimoda, Kenji Kohara, Shuhei Nakanishi, Tomoatsu Mune, Kohei Kaku, Hideaki Kaneto

**Affiliations:** Department of Diabetes, Endocrinology and Metabolism, Kawasaki Medical School, 577 Matsushima, Kurashiki, 701-0192 Japan

**Keywords:** Molecular biology, Endocrinology

## Abstract

There has been no report about the mechanism for anti-atherosclerotic effects of dulaglutide (Dula) and/or about the difference of its effectiveness between in an early and a late phase of diabetes. To address such questions, streptozotocin (STZ) was intraperitoneally injected to ApoE knockout mice at 8 weeks of age. Either Dula or vehicle was administered to STZ-induced diabetic ApoE knockout mice from 10 to 18 weeks of age as an early intervention group and from 18 to 26 weeks as a late intervention group. Next, non-diabetic ApoE knockout mice without STZ injection were subcutaneously injected with either Dula or vehicle. In an early intervention group, atherosclerotic lesion in aortic arch and Mac-2 and CD68-positive areas in aortic root were significantly smaller in Dula group. In abdominal aorta, expression levels of some villain factors were lower in Dula group. In a late intervention group, there were no immunohistological differences in aortic root and expression levels of various factors between two groups. Furthermore, even in non-diabetic ApoE knockout mice, expression levels of inflammatory and macrophage markers were reduced by treatment with Dula. Taken together, Dula exerts more beneficial anti-atherosclerotic effects in an early phase of diabetes rather than in a late phase.

## Introduction

Glucagon-like peptide-1 (GLP-1) is a peptide hormone with anti-inflammatory effects which was first found to regulate insulin secretion from pancreatic β-cells, and agonists of GLP-1 receptor are widely used for the treatment of type 2 diabetes mellitus all over the world^1^. GLP-1 receptor expression is observed not only in the pancreas but also in a variety of tissues including arteries such as endothelial and smooth muscle cells^2^. Recently, several large-scale double-blind, randomized placebo-controlled trials have reported that GLP-1 receptor agonists such as liraglutide (LEADER trial), semaglutide (SUSTAIN-6 trial), albiglutide (Harmony Outcomes), oral semaglutide (PIONEER trial) and dulaglutide (REWIND trial) reduced 3-point major adverse cardiovascular events^3–7^. Therefore, it has been drawn much attention in clinical practice that GLP-1 receptor agonist would be very promising not only for obtaining good glycemic control but also for preventing atherosclerosis and subsequent cardiovascular events. Indeed, positioning of GLP-1 receptor agonists in diabetes therapy is evaluated once more at present. Furthermore, GLP-1 receptor agonists are highly valued when stipulating clinical guidelines for the treatment of diabetes mellitus and its complications^8^.

Among above-mentioned several large-scale clinical trials, there are several notable points in REWIND trial. First, this trial was designed at the beginning to show no non-inferiority to placebo with respect to cardiovascular events. Second, this trial included many primary prevention populations; participants with established cardiovascular disease accounted for only 31.5% in REWIND trial, which was much lower compared with other trials. Third, a median follow-up period was as long as 5.4 years, which was much longer than other trials, and average baseline HbA1c was as low as 7.3%, which was much lower than other trials^7,9^. When considered inclusion criteria of REWIND trial, dulaglutide might be effective for both primary and secondary cardiovascular prevention in a high proportion of people with type 2 diabetes. In addition, dulaglutide reduced cardiovascular events in people with HbA1c similar to those in the other trials with higher baseline HbA1c concentrations (hazard ratio [HR] 0.88, 95% CI 0.79–0.99; *p* = 0.026, non-fatal stroke: HR 0.76, 95% CI 0.61–0.95; *p* = 0.017). When considered the differences of such participants’ baseline characteristics, it raises questions about the mechanism of anti-atherosclerotic effects of dulaglutide in subjects with and without diabetes and the difference of its effectiveness between intervention from an early and a late phase of diabetes. However, there has been no basic study using dulaglutide except for one report from our group, which investigated its effects on pancreatic β-cells^10^. Therefore, we aimed to clarify the mechanism for anti-atherosclerotic effects of dulaglutide and to compare its effectiveness between an early and a late phase of diabetes.

## Results

### Dulaglutide (Dula) improved glycemic control in STZ-induced diabetic ApoE knockout mice in both an early and a late intervention group

**Figure 1:**
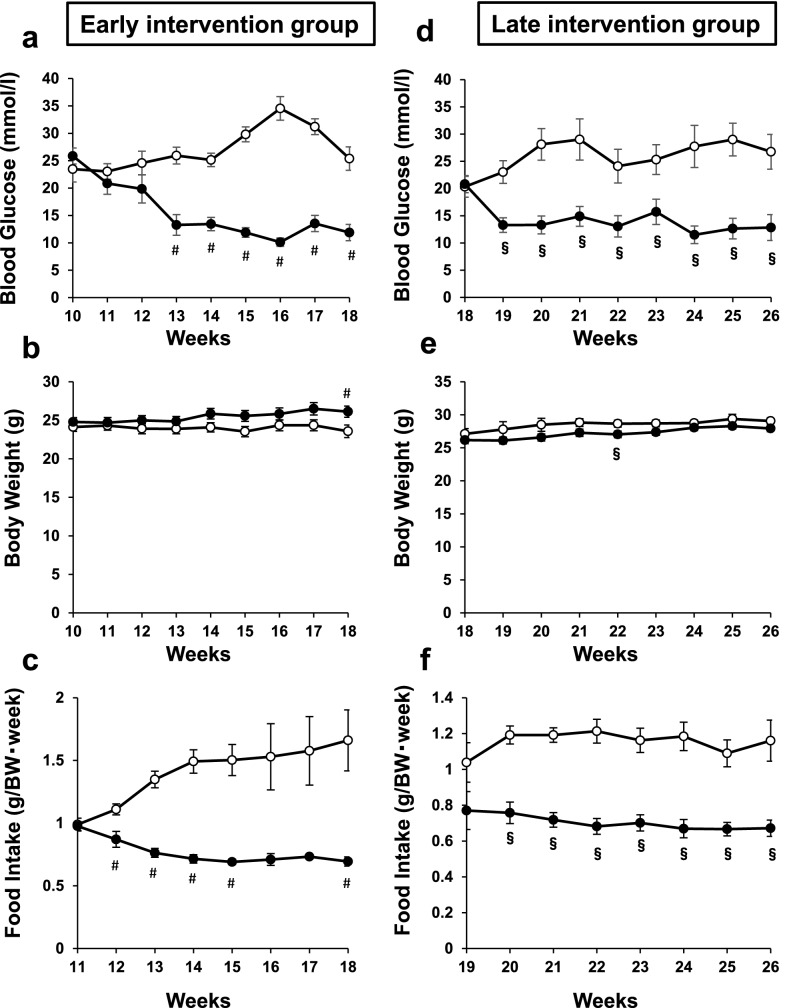
Blood glucose levels, body weights and food intake in an early and a late intervention group in STZ-induced ApoE knockout mice. STZ-induced diabetic ApoE knockout mice were subcutaneously injected either Dula (0.6 mg/kg × 2/week) or vehicle in an early and a late intervention group. (**a**) Blood glucose levels, (**b**) body weights and (**c**) food intake in an early intervention group. Dula (black circle) (n = 9), control (white circle) (n = 9). (**d**) Blood glucose levels, (**e**) body weights and (**f**) food intake in a late intervention group. Dula (n = 8), control (n = 10). Values are the mean ± SEM of data obtained from each group. #*p* < 0.05 (Early Cont vs Early Dula), §*p* < 0.05 (Late Cont vs Late Dula).

Dula significantly improved glycemic control, accompanied by reduced food intake in both an early (Early Cont group vs Early Dula group: 10–18 weeks of age) and a late intervention group (Late Cont group vs Late Dula group: 18–26 weeks of age) (Fig. 1a,c,d,f). At 18 weeks of age, blood glucose levels in Early Cont and Early Dula groups were 25.4 ± 2.14 mmol/L and 11.9 ± 1.49 mmol/L, respectively (*p* < 0.05). At 26 weeks, blood glucose levels in Late Cont and Late Dula groups were 26.8 ± 3.19 mmol/L and 12.8 ± 2.38 mmol/L, respectively (*p* < 0.05). On the contrary, body weight change was comparable between both in Early Cont and Early Dula groups and in Late Cont and Late Dula groups (Fig. 1b,e), probably because the influence of reduced food intake on body weight was offset by the amelioration of glycemic control in Dula group.

There was no difference in systolic and diastolic blood pressure and in heart rate between Early Cont and Early Dula groups (Supplementary Table 1), suggesting that the influence of Dula on blood pressure and heart rate is insignificant. Lipid profiles (total cholesterol, triglyceride, HDL cholesterol, LDL cholesterol and non-esterified fatty acid (NEFA)) were substantially ameliorated in Early Dula group compared to Early Cont group. At 18 weeks of age, lipid profiles in Early Cont and Early Dula groups were as follows: total cholesterol, 17.1 ± 1.32 vs 10.6 ± 1.34 mmol/L; triglyceride, 3.3 ± 0.45 vs 2.1 ± 0.37 mmol/L; HDL cholesterol, 1.77 ± 0.13 vs 1.13 ± 0.09 mmol/L; LDL cholesterol, 6.36 ± 0.55 vs 4.02 ± 0.31 mmol/L; NEFA, 3.37 ± 0.27 vs 2.45 ± 0.20 mEq/L (Supplementary Table 1). Among them, the difference in serum total cholesterol, HDL cholesterol, LDL cholesterol and NEFA reached statistical significance. Lipid profiles were also substantially ameliorated in Late Dula group compared to Late Cont group. At 26 weeks of age, lipid profiles in Late Cont and Late Dula groups were: total cholesterol, 12.0 ± 1.62 vs 8.6 ± 1.27 mmol/L; triglyceride, 2.5 ± 0.35 vs 1.5 ± 0.22 mmol/L; HDL cholesterol, 1.23 ± 0.13 mmol/L vs 0.83 ± 0.08 mmol/L; LDL cholesterol, 3.98 ± 0.32 mmol/L vs 3.29 ± 0.20 mmol/L; NEFA, 2.90 ± 0.18 vs 2.04 ± 0.10 mEq/L respectively (Supplementary Table 2). Among them, the difference in serum triglyceride, HDL cholesterol and NEFA reached statistical significance. As a whole, lipid profile changes were similar between an early and a late intervention group. Taken together, Dula significantly improved glycemic control in STZ-induced diabetic ApoE knockout mice together with substantial amelioration of lipid profiles both in Early Dula and Late Dula groups compared to their control groups. Furthermore, we also administered Dula to non-diabetic ApoE knockout mice from 10 to 18 weeks (non-DM Cont group vs non-DM Dula group). At the end of treatment, there were no differences in blood glucose levels, body weight, total cholesterol, triglyceride, HDL cholesterol, LDL cholesterol and NEFA between non-DM Cont and non-DM Dula groups (Supplementary Table 3).

### Plaque formation in aortic arch was significantly reduced by Dula treatment in an early intervention group and a late intervention group, while there was no difference in Masson trichrome positive area and necrotic core size

**Figure 2:**
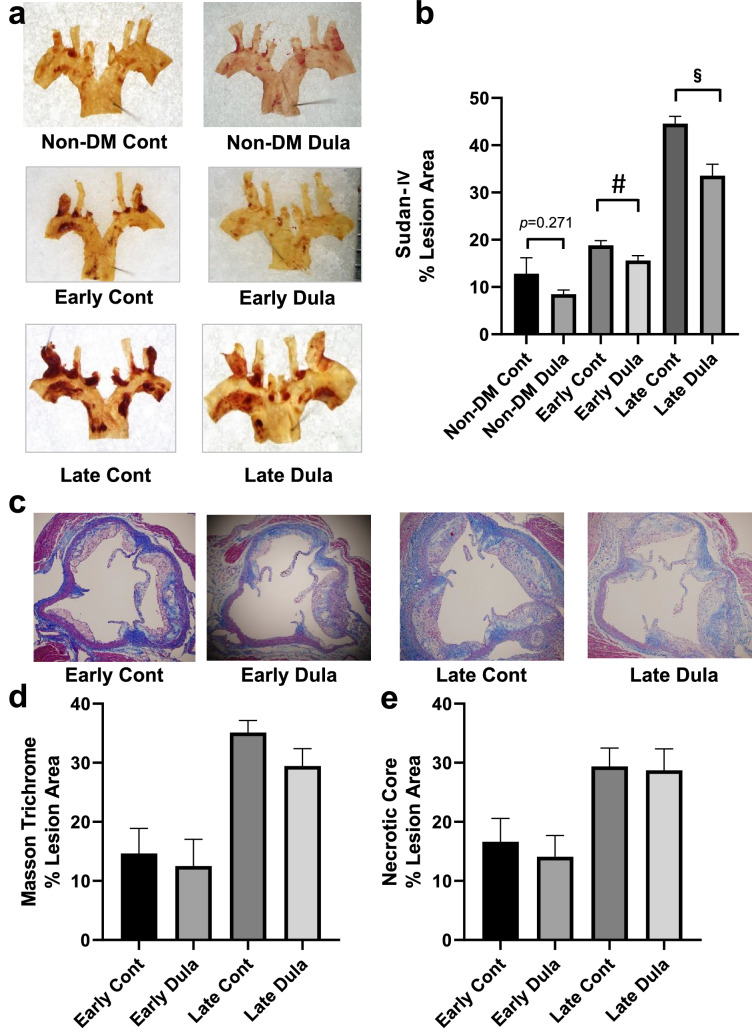
Atherosclerotic lesion in aortic arch and Masson trichrome staining of aortic root in ApoE knockout mice. (**a**) Representative Sudan-IV staining pictures in aortic arch. (**b**) Sudan-IV positive area (% lesion area) (**c**) Representative Masson trichrome staining pictures in aortic root. (**d**) Masson trichrome staining (% lesion area). (**e**) Necrotic core in aortic root (% lesion area). Non-DM Cont (n = 7), non-DM Dula (n = 6), Early Cont (n = 5–6), Early Dula (n = 5 ~ 6), Late Cont (n = 6), Late Dula (n = 6). Values are the mean ± SEM of data obtained from each group. #*p* < 0.05 (Early Cont vs Early Dula), §*p* < 0.05 (Late Cont vs Late Dula).

Next, to examine the possible effects of Dula on atherosclerosis, we evaluated plaque formation in the aortic arch and conducted immunohistological analyses of aortic root such as CD68, Mac-2 and Masson Trichrome staining. As a result, Sudan IV staining revealed significantly reduced plaque area in the aortic arch in both Early and Late Dula groups compared to their control groups (*p* < 0.05), while it did not reach statistical significance between non-DM Dula group and non-DM Cont group (*p* = 0.271) (Fig. 2a,b). There was no big difference in Masson Trichrome staining in aortic root between Dula and Cont groups (Fig. 2c,d). To further investigate plaque stability, necrotic core was evaluated in aortic root, which resulted in no difference between Dula and Cont groups (Fig. 2e). Taken together, these data suggest that Dula exerts significant anti-atherosclerotic effects in diabetic mice.

### Mac-2 and CD68-positive areas in aortic root were significantly reduced by Dula treatment in an early intervention group but not in a late intervention group

**Figure 3:**
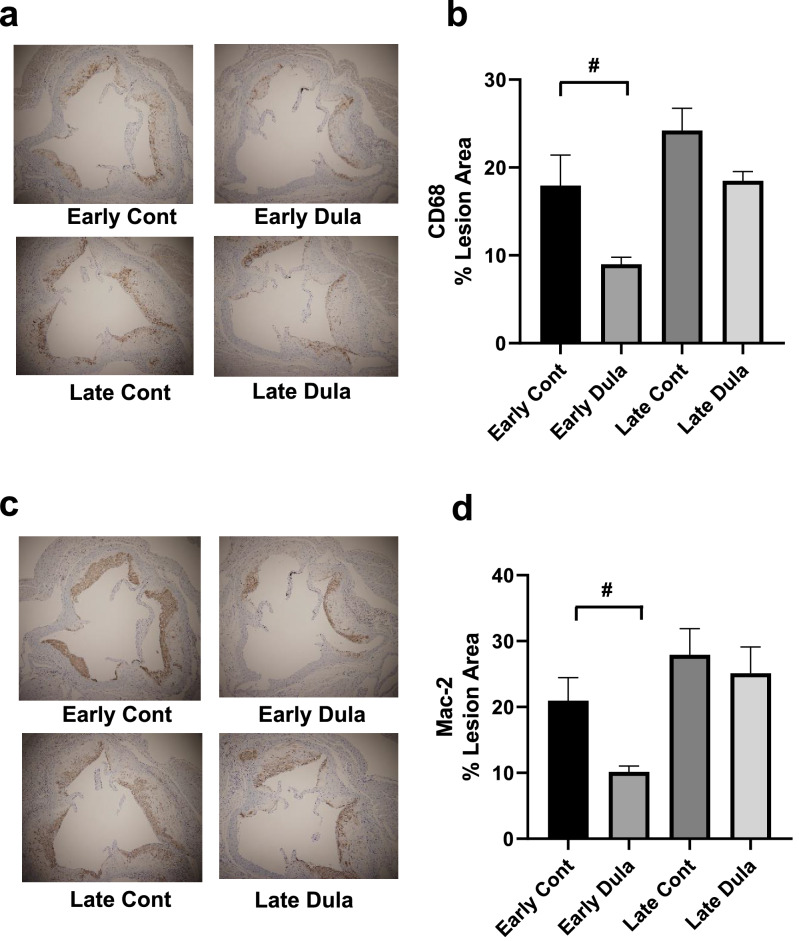
CD68 and Mac2 staining of aortic root in early and late intervention groups in STZ-induced ApoE knockout mice. (**a**) Representative CD68 staining pictures, (**b**) CD68 positive area (% lesion area), (**c**) Representative Mac2 staining pictures, (**d**) Mac2 positive area (% lesion area). Early Cont (n = 5–6), Early Dula (n = 5–6), Late Cont (n = 6), Late Dula (n = 6). #*p* < 0.05 (Early Cont vs Early Dula).

We next evaluated macrophage infiltration in the plaque of aortic root. Interestingly, immunohistological analyses of aortic root presented significantly reduced Mac-2 and CD68-positive areas in Early Dula group compared to Early Cont group (*p* < 0.05) (Fig. 3a–d). On the other hand, no significant difference was observed in a late intervention group (Fig. 3a–d), which suggested early intervention is more effective than late intervention in the aspect of macrophage infiltration into plaque lesion.

### Expression levels of inflammatory and coagulation markers were significantly reduced by Dula treatment in an early intervention group accompanied with recovery of *Glp-1r* expression level in abdominal aorta

**Figure 4:**
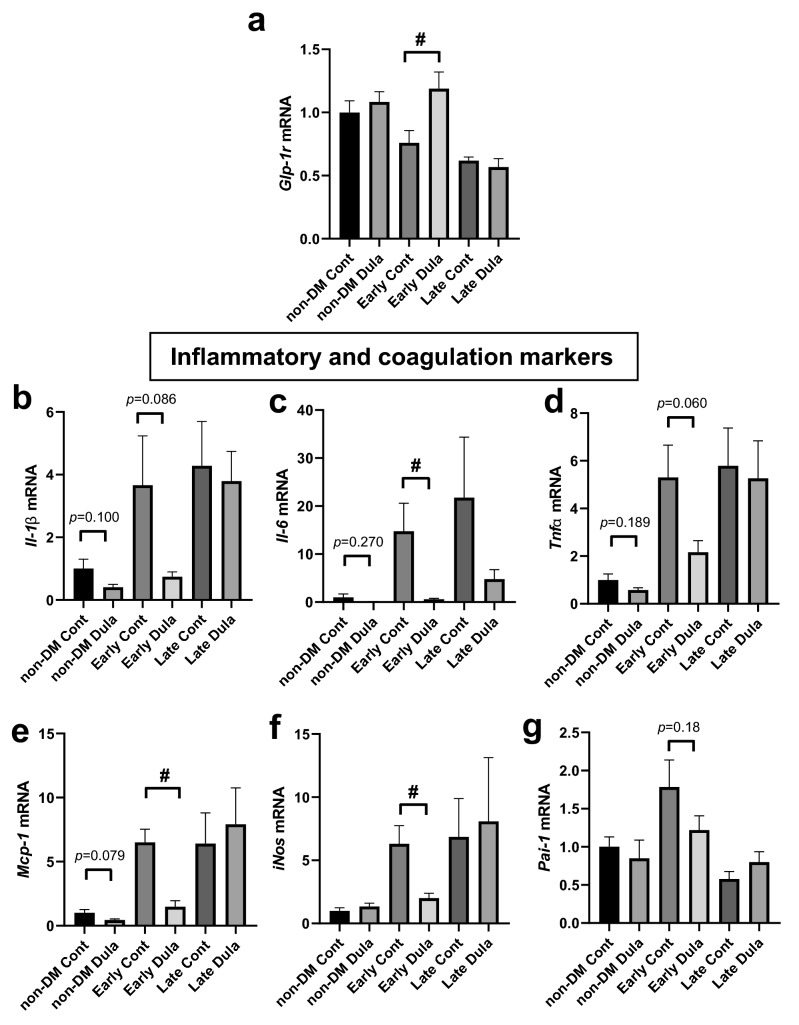
mRNA expression levels of *Glp-1r* and inflammatory and coagulation markers in abdominal aorta. (**a**) *Glp-1r* mRNA expression levels, (**b**–**g**) mRNA expression levels of inflammatory and coagulation markers expressed as fold change relative to non-diabetic control group. Non-diabetic Cont (n = 8), Non-diabetic Dula (n = 8), Early diabetic Cont (n = 8), Early diabetic Dula (n = 6), Late diabetic Cont (n = 8), Late diabetic Dula (n = 7). Values are the mean ± SEM of data obtained from each group. #*p* < 0.05 (Early Cont vs Early Dula).

To further examine the protective effects of Dula against atherosclerosis, we evaluated mRNA expression levels of *Glp-1r,* inflammatory and coagulation markers in abdominal aorta. Interestingly, mRNA expression of *Glp-1r* was significantly higher in Early Dula group compared to Early Cont group (*p* < 0.05) (Fig. 4a), probably due to alleviation of glucose toxicity by treatment with Dula. Expression levels of *Il-6*, *Mcp-1* and *iNos* were significantly lower in Early Dula group compared to Early Cont group (*p* < 0.05). (Fig. 4c,e,f), while such significant difference was not observed between Late Cont and Late Dula groups. Expression levels of *Il-1β* and *Tnf-α* were also substantially lower in non-DM Dula and Early Dula groups compared to their controls, although it did not reach statistical significance (Fig. 4b,d). Plasminogen activator inhibitor (*Pai1*) tended to be lower in Early Dula group compared to Early Cont group, which did not reach statistical significance (Fig. 4g). These data suggested that *Glp-1r* expression level in Early Dula group become comparable to non-DM group. Furthermore, in a late treated group, *Glp-1r* expression levels were lower and Dula did not succeed in the recovery of its expression in a late intervention group. In addition, inflammatory markers were substantially or significantly lower in non-DM Dula and Early Dula groups, while such tendency was not observed in a late intervention group.

### mRNA expression levels related to cell adhesion in abdominal aorta were substantially or significantly reduced in Dula treatment in non-DM and early intervention groups, while those related to plaque stability were relatively comparable among all groups

**Figure 5:**
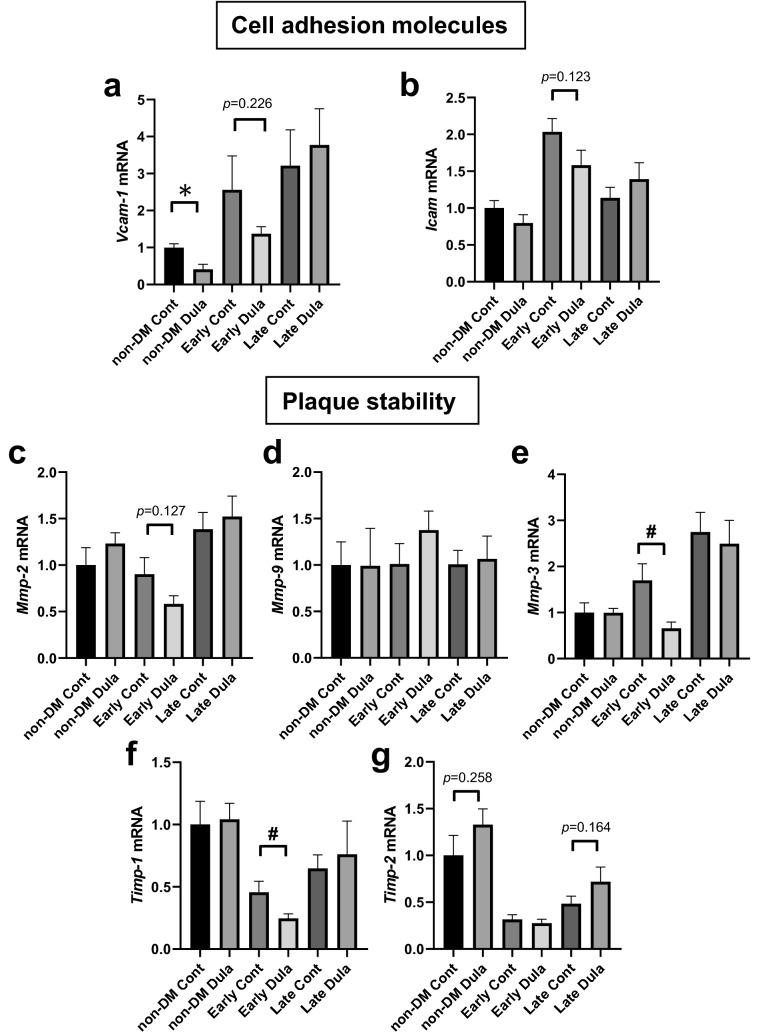
mRNA expression levels related to cell adhesion molecules and plaque stability in abdominal aorta. (**a**, **b**) mRNA expression levels related to cell adhesion molecules such as *Vcam-1* and *Icam*. (**c**–**g**) mRNA expression levels related to plaque stability such as *Mmp-2, Mmp-9, Mmp-3, Timp-1 and Timp2* expressed as fold change relative to non-diabetic control group. Non-diabetic Cont (n = 8), Non-diabetic Dula (n = 8), Early diabetic Cont (n = 8), Early diabetic Dula (n = 6), Late diabetic Cont (n = 8), Late diabetic Dula (n = 7). Values are the mean ± SEM of data obtained from each group. *p < 0.05 (non-DM Cont vs non-DM Dula), #*p* < 0.05 (Early Cont vs Early Dula).

Next, we evaluated mRNA expression levels related to cell adhesion molecules and plaque stability in abdominal aorta. *Vcam-1* expression level was significantly reduced in non-DM Dula group and tended to be reduced in Early Dula group (*p* = 0.226) (Fig. 5a). In *Icam* expression, there was no significant difference although tendency was observed in Early group (*p* = 0.123) (Fig. 5b). To assess plaque stability, we also explored mRNA expression levels of matrix metalloproteinases such as *Mmp-2*, *Mmp-9 and Mmp-3*, which are related to plaque instability. Expression levels of *Mmp-2* and *Mmp-3* were substantially or significantly lower in Early Dula group, while no significant difference was observed in *Mmp-9* (Fig. 5c–e). Furthermore, we also evaluated mRNA expression levels of tissue inhibitors of metalloproteases such as *Timp-1*and *Timp-2*, which are natural inhibitors of Mmps. *Timp-1* was significantly lower in Early Dula group, while *Timp-2* showed tendency to increase in Dula-treated non-DM and late intervention group (*p* = 0.258 and *p* = 0.164) (Fig. 5f,g).

### mRNA expression levels of macrophage markers in abdominal aorta were significantly reduced in Dula-treated early intervention group, while M2-like macrophage markers were comparable except for a late intervention group

**Figure 6:**
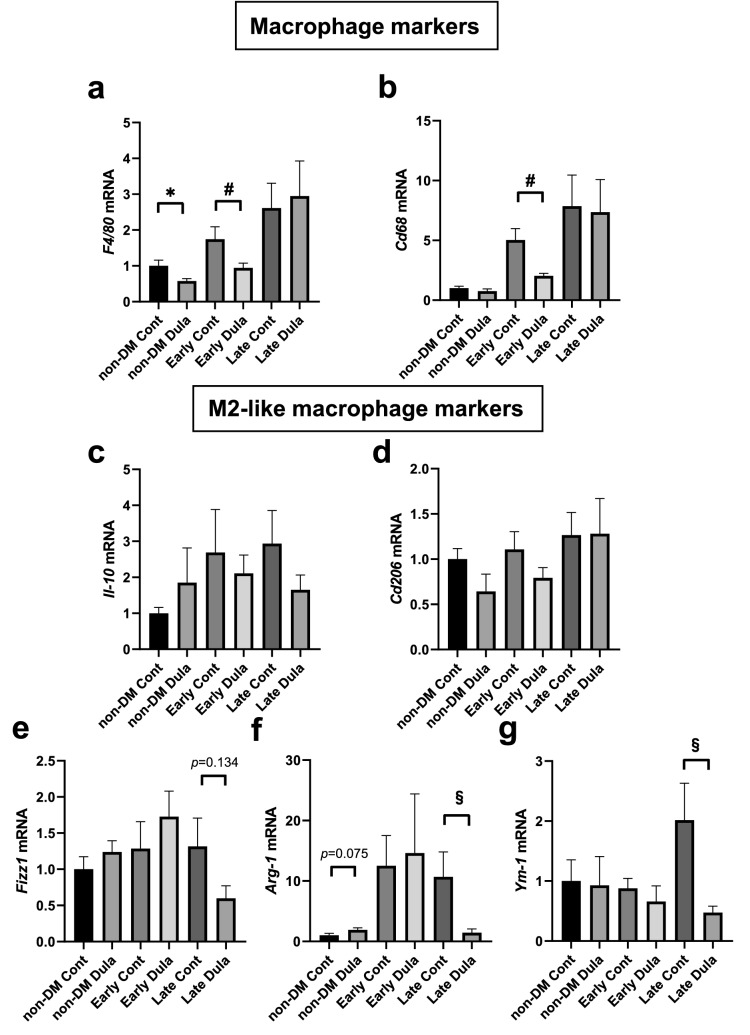
mRNA expression levels of macrophage markers and M2-like macrophage markers in abdominal aorta. (**a**, **b**) mRNA expression levels of macrophage markers such as *F4/80* and *Cd68*. (**c**–**g**) mRNA expression levels M2-like macrophage markers such as *Il-10, Cd206, Fizz1, Arg-1* and *Ym-1* expressed as fold change relative to control group. Dula (n = 7), Control (n = 8). *p < 0.05 (non-DM cont vs non-DM Dula), #*p* < 0.05 (Early Cont vs Early Dula), §*p* < 0.05 (Late Cont vs Late Dula).

mRNA expression level of *F4/80* presented gradually increase in Cont groups (non-DM Cont < Early Cont < Late Cont), which was significantly reduced in non-DM Dula and Early Dula groups (*p* < 0.05), although there was no such tendency in Late Dula group (Fig. 6a). mRNA expression level of *Cd68* showed similar features to *F4/80* expression although it did not reach significancy in non-DM Cont group (Fig. 6b).

As we confirmed significant or substantial decreases in M1-like macrophage markers such as *Il-1β*, *Il-6*, *Tnfα*, *Mcp-1* and *iNos* in non-DM Dula and Early Dula groups (Fig. 4b–f), we next focused on M2-like macrophage markers in abdominal aorta. As shown in Fig. 6c,d, there were no differences in mRNA expression levels of *Il-10* and *Cd206*. mRNA expression level of *Fizz1* (so called *Relm-α*) presented substantial decrease in Late Dula group (*p* = 0.134) (Fig. 6e). Interestingly, arginase-1 (*Arg-1*) was significantly lower in Late Dula group even though there was no change in Early Dula group, which was completely opposite result to *iNos* expression level (Fig. 6f). *Ym-1* expression level was significantly lower in Late Dula group (Fig. 6g).

## Discussion

This is the first study elucidating that dulaglutide exerts anti-atherosclerotic effects in STZ-induced diabetic ApoE knockout mice. Importantly, altered expression levels of vascular factors related to chemoattractant, inflammation and macrophage infiltration in abdominal aorta strongly suggest that dulaglutide would exert more beneficial anti-atherosclerotic effects in an early phase of diabetes rather than in a late phase in the long run. Moreover, dulaglutide also presented beneficial vascular protective effects in non-diabetic ApoE knockout mice without STZ treatment, in which there were no differences in blood glucose levels, body weights and lipid profiles between Dula-treated and control group. These results suggest that dulaglutide exerts some anti-atherosclerotic effects in a glucose-independent manner in addition through its glucose-lowering effects.

In this study, plaque formation in aortic arch was significantly reduced in both an early and a late intervention group. These results might be partially associated with the findings that in REWIND trial, dulaglutide was effective for both primary and secondary cardiovascular prevention. However, the mechanism for amelioration of plaque formation might be different between an early and a late intervention group. Indeed, although amelioration of glycemic control by Dula treatment largely contributed to the decrease of plaque formation in both groups, improvements in expression levels of various vascular factors were observed only in an early intervention group. It has been reported that expression levels of incretin receptors in pancreatic β-cells are reduced under diabetic conditions but preserved after alleviation of glucose toxicity with some anti-diabetic drugs^11–14^. In this study, *Glp-1r* expression level in arteries was preserved in Dula-treated group in an early intervention group which was presumably due to alleviation of glucose toxicity by treatment with Dula. In contrast, there was no difference in *Glp-1r* expression levels between Dula-treated and control group in a late intervention group, although glucose levels and lipid profiles were substantially improved. We assume that the term of exposure to glucose toxicity was too long for vascular *Glp-1r* expression to be recovered in a late intervention group. It still needs further investigation to address precise mechanism of this result.

In vitro experiments, Hattori et al*.* demonstrated that liraglutide downregulated *Tnf-α*, *Vcam1* and *Icam1*in human umbilical vein endothelial cells^15^. Similar in vivo experiments were performed in ApoE knockout mice treated with GLP-1 analog, where exendin-4 reduced numbers of Mac-2-positive cells adhering to the endothelium and decreased levels of *Vcam-1* and *Icam-1* mRNA expressions^16^. It was also reported that GLP-1 receptor stimulation increased the expression of eNOS and suppressed the intercellular adhesion molecule-1 (*Icam-1*) expression of aortic endothelial cells in arteriosclerosis model mice^17^. In other studies, it has been reported that liraglutide promotes anti-inflammatory responses^18–20^, reduces foam cell formation^21,22^, inhibits expression of inflammatory markers and monocyte adhesion and attenuates atherosclerosis in vivo^23,24^. It has been also reported that liraglutide improves plaque stability^17^. In our study, mRNA expression level of *Mmp3* was significantly reduced in an early intervention group accompanied with decrease of *Timp1* expression level. Although no significant difference was observed in necrotic core in this study, mRNA expression changes of *Mmp3* and *Timp1* might suggest plaque stabilization especially in an early intervention group which agrees with the previous report^25^. The results of these previous in vivo and in vitro studies agree with the results of an early intervention group and non-diabetic group in our study. However, it is reported that internalization and recycling of GLP-1 receptor in pancreatic β-cells is different among various GLP-1RAs. If the same phenomenon exists in vascular endothelial or smooth muscle cells, we cannot completely deny drug effects of each GLP-1 receptor agonist^26^.

It is well know that cholesterol levels influence the progression of atherosclerosis. In our study, both LDL and HDL cholesterol levels were reduced by dulaglutide treatment in diabetic mice by some unknown reason. Therefore, it is difficult to evaluate how alteration of cholesterol levels influenced the progression of atherosclerosis. Further study would be necessary to clarify the mechanism how dulaglutide alters cholesterol levels.

There were several reports showing anti-atherosclerotic effects of GLP-1RAs especially by using liraglutide^17,25,27^. We think, however, that there are several new findings in this study. First, dulaglutide has drawn much attention and is very often used in clinical practice due to its durability of glucose-lowering effect and its usefulness even in lean subjects, whereas liraglutide has stronger effects on body weight reduction. In such a situation, in this study we evaluated the anti-atherosclerotic effects of dulaglutide and compared such effects between in an early and a late stage, whereas several previous studies were performed using liraglutide. Second, in this study we used diabetic as well as non-diabetic mouse model, and evaluated anti-atherosclerotic effects of dulaglutide in both models, whereas it was reported that liraglutide inhibited the progression of early onset and low-burden atherosclerotic disease in non-diabetic mouse model^17^. Finally and importantly, in this study we showed a possible underlying mechanism for anti-atherosclerotic effects of dulaglutide especially in an early stage. The data in this study indicate that recovery of incretin receptor expression and reduction of macrophages infiltration and inflammation by dulaglutide treatment explains, at least in part, the mechanism for its anti-atherosclerotic effects.

It remains unknown why dulaglutide did not reduce atherosclerotic plaque in non-diabetic ApoE-knockout mice but reduced it in diabetic ApoE-knockout mice in this study which point was different from the findings in recent study with other GLP-1 analogs liraglutide or semaglutide^25^. It is thought that GLP-1RAs exert anti-atherosclerotic effects in GLP-1R-dependent as well as GLP-1R-independent manner. Although speculative, we think there is a possibility that other GLP-1RAs such as liraglutide or semaglutide have stronger GLP-1-dependent anti-atherosclerotic effects compared to dulaglutide. To clarify this point, it would be necessary to directly compare such effects among these three GLP-1RAs. In addition, it remains unknown how dulaglutide treatment reduced aortic plaque area without affecting mRNA levels of atherosclerosis-related molecules in the late intervention experiment. It seems that there are various factors which could influence aortic plaque area in addition to those we examined in this study. Also, we cannot deny the possibility that this result might derive from glucose-lowering effects or changes of lipid profile with dulaglutide treatment. Oppositely, it also remains unknown why dulaglutide failed to reduce aortic plaque area with ameliorated mRNA levels of such molecules in the non-diabetic mice. We assume that reduction of aortic plaque area requires some alteration in protein levels and/or post-translational modification in addition to alteration of mRNA levels.

There are several limitations in this study. First, the number of used animals in this study is not very large for this kind of studies to get firm conclusions. Second, in this study we failed to evaluate the possible change in smooth muscle cells by dulaglutide treatment, although we evaluated macrophage phenotype. Third, since we used the whole abdominal aorta, it is difficult to distinguish in which cells dulaglutide provides its effectiveness such as myeloid derived macrophage and neutrophil, endothelial cells and smooth muscle cells. Recently, Helmstädter et al*.* reported that in hypertensive rodent model, liraglutide treatment normalized blood pressure, cardiac hypertrophy, vascular fibrosis, endothelial dysfunction, oxidative stress and vascular inflammation in an endothelial GLP-1R-dependent and myeloid cell GLP-1R-independent manner^28^. This report suggests that in our model, it is possible that reduction of plaque formation was in an endothelial GLP-1R-dependent manner.

Taken together, dulaglutide exerts anti-atherosclerosis effects in a glucose-independent manner as well as through its glucose-lowering effects. In addition, when we use dulaglutide in an early phase of diabetes, more beneficial anti-atherosclerotic effects would be expected, especially in the aspect of recovery of incretin receptor expression and reduction of macrophages infiltration and inflammation by dulaglutide treatment. Therefore, we propose that it would be better to use dulaglutide in an earlier phase of diabetes in clinical practice from the point of cardiovascular protection.

## Materials and methods

### Animals and diets

ApoE knockout mice (C57BL/6J-ApoEtm1Unc) were purchased from Charles River Laboratories and housed (two animals per cage for all experiments) under controlled ambient conditions and 12 h light and dark cycle. The animals were given free access to water and standard diet (MF; Oriental Yeast Co., Ltd.) and maintained at 25 °C.

They were intraperitoneally injected with streptozotocin (STZ: FUJIFILM Wako Pure Chemical Corporation, Japan) (50 mg/kg/day) for 5 consecutive days at 8 weeks of age. When mice showed apparent hyperglycemia superior to 300 mg/dL at fed status, we diagnosed them as having diabetes. ApoE knockout mice with diabetes were divided into two groups: one group received dulaglutide (Dula: purchased from Eli Lilly, Japan) subcutaneously (0.6 mg/kg twice weekly) as we previously reported^10^. The other group received vehicle subcutaneously (phosphate-buffered saline twice weekly) from 10 to 18 weeks of age as an early intervention group or from 18 to 26 weeks of age as a late intervention group.

In addition, to exclude the influence of glucose-lowering effects of Dula, we investigated the effect of Dula on non-diabetic ApoE knockout mice without STZ injection from 10 to 18 weeks of age. Body weight and food intake were measured during the experiments.

The study was approved by the animal use committee of Kawasaki Medical School (No. 18-031) and conducted in compliance with the animal use guidelines of Kawasaki Medical School.

### Measurement of biochemical markers

Blood samples were collected from tail vein. Blood glucose levels were measured using a glucometer (Glutest Mint; Sanwa Kagaku Kenkyusho Co, Ltd, Japan). Plasma total cholesterol, LDL cholesterol, HDL cholesterol, triglyceride and non-esterified fatty-acid (NEFA) were measured enzymatically using the Wako LabAssay, E-Test and C-Test (Wako Pure Chemical Industries, Japan).

### Blood pressure measurement

Blood pressure was measured at the beginning and the end of the intervention. Measurement was performed using a computerized non-invasive tail-cuff system (NP-NIBP MONITOR FOR MICE & RATS, MUROMACHI KIKAI Co, Ltd, Japan). Animals were placed in mice holder and blood pressure was measured 3 times per mouse. The average of 3 measurements was presented as data.

### RNA isolation and real time PCR

Total RNA was extracted from abdominal aorta using an RNeasy lipid tissue mini kit (QIAGEN, Valencia, CA) according to the manufacturer's instructions. cDNA was produced from mRNA using TaqMan reverse transcription reagents (Applied Biosystems, Foster City, CA). Quantitative RT-PCR was conducted using a 7500 Real-Time PCR system (Applied Biosystems). To quantify gene expression, the 2^−ΔCT^ was calculated using β-actin as an internal control. Primer sequences used for real time PCR are presented in supplementary Table 4.

### Histological and immunohistological analyses

Under anesthesia, PBS was perfused from left ventricle and then animals were killed and heart and aorta were dissected. Sudan IV (Wako: 192-04392) staining was conducted for aortic arch. Adventitial fat tissue was removed and aorta was dissected longitudinally. The image analysis software NIH Image (version 1.61; http://rsbweb.nih.gov/ij/) was used to calculate the ratio of the plaque lesion to the total aortic arch area.

Isolated heart was fixed overnight with formalin at 4 °C. Tissue was routinely processed for paraffin embedding and 4 μm sections of aortic root were cut and mounted on silanised slides and were stained by Masson trichrome, CD68 (ab125212, 1:500) and Mac-2 (CL8942AP, 1:2000). The positive areas were calculated as the ratio of the positively stained area to the total aortic valve area. Necrotic core areas, defined as areas primarily composed of lipids with cholesterol clefts, cellular debris, and foam cells, were determined from Masson trichrome stained sections.

### Statistical analysis

All data were analyzed and expressed as the mean ± standard error of the mean. In this study, differences between two groups such as non-DM Cont vs non-DM Dula (*), Early Cont vs Early Dula (#) and Late Cont vs Late Dula (§) were tested for statistical significance using Student’s t-test. *p* values less than 0.05 were considered to indicate a statistically significant difference.

## Supplementary Information

Supplementary Tables.

## Data Availability

The datasets used and/or analyzed in the current study are available from the corresponding authors upon reasonable request.
