# Supplementary material for: Dulaglutide exerts beneficial anti atherosclerotic effects in ApoE knockout mice with diabetes: the earlier, the better
Source: Sci Rep. 2021 Jan 14;11:1425. doi: 10.1038/s41598-020-80894-x (PMC7809053; doi:10.1038/s41598-020-80894-x)
Supplement: Supplementary file 1 — Supplementary Tables. [file 41598_2020_80894_MOESM1_ESM.pdf]

Supplementary Information

Dulaglutide exerts beneficial anti atherosclerotic effects in ApoE knockout mice with diabetes: The earlier, the better

Junpei Sanada, Atsushi Obata＊, Yoshiyuki Obata, Yoshiro Fushimi, Masashi Shimoda, Kenji Kohara, Shuhei Nakanishi, Tomoatsu Mune, Kohei Kaku, Hideaki Kaneto＊

Department of Diabetes, Endocrinology and Metabolism,
Kawasaki Medical School

Supplementary Table 1

Biochemical data, body weights, and blood pressure in early intervention group

	Before treatment (10 weeks)		After treatment (18 weeks)	
	Control	Dulaglutide	Control	Dulaglutide
Blood glucose (mmol/l)	23.5±2.36	25.9±1.46	25.4±2.14	11.9±1.49#
Total cholesterol (mmol/l)	N/A	N/A	17.1±1.32	10.6±1.34#
Triglyceride (mmol/l)	N/A	N/A	3.3±0.45	2.1±0.37
HDL cholesterol (mmol/l)	N/A	N/A	1.77±0.13	1.13±0.09#
LDL cholesterol (mmol/l)	N/A	N/A	6.36±0.55	4.02±0.31#
NEFA (mEq/l)	N/A	N/A	3.37±0.27	2.45±0.20#
Body weights (g)	24.1±0.56	24.8±0.57	23.6±0.82	26.1±0.72#
Systolic BP (mmHg)	114.1±11.2	101.0±6.03	97.1±3.13	100.6±4.19
Diastolic BP (mmHg)	61.2±9.14	59.1±8.11	49.1±7.17	63.7±3.62
Heart rate (beats/min)	650.5±27.5	608.5±30.2	663.6±42.3	666.8±20.7

NEFA, non-esterified fatty acid; BP, blood pressure; #: $p<0.05$ (Early Cont vs Early Dula)

Supplementary Table 2：Biochemical data and body weights in late intervention group

	Before treatment (18 weeks)		After treatment (26 weeks)	
	Control	Dulaglutide	Control	Dulaglutide
Blood glucose (mmol/l)	20.3±1.93	20.8±1.57	26.8±3.19	12.8±2.38 §
Total cholesterol (mmol/l)	N/A	N/A	12.0±1.62	8.6±1.27
Triglyceride (mmol/l)	N/A	N/A	2.5±0.35	1.5±0.22 §
HDL cholesterol (mmol/l)	N/A	N/A	1.23±0.13	0.85±0.08 §
LDL cholesterol (mmol/l)	N/A	N/A	3.98±0.32	3.29±0.20
NEFA (mEq/l)	N/A	N/A	2.90±0.18	2.04±0.10 §
Body weights (g)	27.1±0.81	26.2±0.54	29.1±0.51	27.9±0.48

NEFA, non-esterified fatty acid; §: p<0.05 (Late Cont vs Late Dula)

Supplementary Table 3：Biochemical data and body weights in non-diabetic group at 18 weeks

	Control	Dulaglutide	*p*
Blood glucose (mmol/l)	9.17±0.52	8.8±0.70	n.s.
Total cholesterol (mmol/l)	7.3±0.64	7.0±1.10	n.s.
Triglyceride (mmol/l)	1.6±0.16	1.2±0.14	n.s.
HDL cholesterol (mmol/l)	0.94±0.15	0.94±0.10	n.s
LDL cholesterol (mmol/l)	3.82±0.48	2.92±0.34	n.s.
NEFA (mEq/l)	1.509±0.135	1.497±0.095	n.s.
Body weights (g)	30.3±0.717	29.4±0.846	n.s.
Systolic BP (mmHg)	98.1±2.96	109.4±4.48	n.s.
Diastolic BP (mmHg)	51.6±5.40	68.5±5.20	n.s.
Heart rate (beats/min)	717.5±8.60	731.1±5.53	n.s.

NEFA, non-esterified fatty acid; n.s., not significant

Supplementary Table 4：Primer sequences of forward and reverse primers for real-time PCR

Genes	Forward	Reverse
β-actin	CGTGAAAAGATGACCCAGATCA	CACAGCCTGGATGGCTACGTA
Glp-1r	ACTTTCTTTCTCCGCCTTGGT	CCTGGTGCAGTGCAAGTGTCT
Mcp-1	CTTCCTCCACCACCATGCA	CCAGCCGGCAACTGTGA
Il-1β	TGGTGTGTGACGTTCCCATTA	CGACAGCACGAGGCTTTTTT
Il-6	ACAACCACGGCCTTCCCTA	CATGTGTAATTAAGCCTCCGACTTG
Tnf-α	TGATCCGCGACGTGGAA	ACCGCCTGGAGTTCTGGAA
Pai-1	TGCATCGCCTGCCATTG	CTTGAGATAGGACAGTGCTTTTTCC
Vcam-1	GATCTCCCCTGAATACAAAACGAT	GCCCGTAGTGCTGCAAGTG
Icam-1	TCGGAAGGGAGCCAAGTAACT	CGACGCCGCTCAGAAGAA
Mmp-9	TATTTTTGTGTGGCGTCTGAGAA	GAGGTGGTTTAGCCGGTGAA
F4/80	TGCATCTAGCAATGGACAGC	GCCTTCTGGATCCATTTGAA
Cd68	TTTCTCCAGCTGTTCACCTTGA	CCCGAAGTGTCCCTTGTCA
Il-10	CAGCCGGGAAGACAATAACTG	CCGCAGCTCTAGGAGCATGT
Cd206	CAAGGAAGGTTGGCATTTGT	CCTTTCAGTCCTTTGCAAGC
inos	GTGACGGCAAACATGACTTCA	GCCATCGGGCATCTGGTA
Arg-1	ACAAGACAGGGCTCCTTTCA	AGCAAGCCAAGGTTAAAGCC
Ym-1	CCATGGCCAAGCTCATTCTT	TCCCTTCTATTGGCCTGTCC
Fizz	ATGAACAGATGGGCCTCCTG	CCCAAGATCCACAGGCAAAG
Mmp-2	CCCTCAAGAAGATGCAGAAGTTC	TCTTGGCTTCCGCATGGT
Mmp-3	ACTCTACCACTCAGCCCAAGG	TCCAGAGAGTTAGACTTGGTGG
Timp-1	GCATGGACATTTATTCTCCACTGT	TCTCTAGGAGCCCCGATCTG
Timp-2	TTCCGGGAATGACATCTATGG	GGGCCGTGTAGATAAACTCGAT
